# Supplementary material for: Smokers’ and Non-Smokers’ Attitudes towards Smoking Cessation in Saudi Arabia: A Systematic Review
Source: Int J Environ Res Public Health. 2020 Nov 6;17(21):8194. doi: 10.3390/ijerph17218194 (PMC7664210; doi:10.3390/ijerph17218194)
Supplement: Supplementary file 1 [file ijerph-17-08194-s001.pdf]

Supplementary Table S1: CEBM critical appraisal tool applied generically

**Author(s) & year** / **Criteria**	Salih & Farghaly 1996[9]	Al-Mohrej et al 2014[27]	Almutairi 2014a[15]	Al-Zalabani et al 2015[10]	Alyamani et al 2016[28]	Baig et al 2016[13]	Hajjar et al 2016[29]	Almogbel et al 2016[30]	Abdelwahab et al 2016[21]	Mahdi et al 2018[31]	Onezi et al 2018[32]	Jradi & Saddik 2018[19]	Alqurashi et al 2019[34]	Al-Nimr et al 2020[35]	Amin et al 2020[36]
1 Did the study address a clearly focused question / issue?	Yes	Yes	Yes	Yes	Yes	Yes	Yes	Yes	Yes	Yes	Yes	Yes	Yes	Yes	Yes
2 Is the research method (study design) appropriate for answering the research question?	Yes	Yes	Yes	Yes	Yes	Yes	Yes	Yes	Yes	Yes	Yes	Yes	Yes	Yes	Yes
3 Is the method of selection of the subjects (employees, teams, divisions, organisations) clearly described?	Yes	No	Yes	Yes	Yes	Yes	Yes	Yes	Yes	Yes	Yes	No	Yes	Yes	Yes
4 Could the way the sample was obtained introduce (selection) bias?	No	Yes	No	No	No	Yes	No	No	No	No	No	Yes	No	No	No
5 Was the sample of subjects representative with regard to the population to which the	Yes	No	Yes	Yes	Unclear	Unclear	Yes	Yes	No	Yes	Yes	No	Yes	Yes	Yes

findings will be referred?															
6 Was the sample size based on pre-study considerations of statistical power?	No	Unclear	No	No	No	No	No	No	No	Yes	No	No	No	No	Yes
7 Was a satisfactory response rate achieved?<br><br>NR=not reported	n=326 (NR)	n=2160 (NR)	n=4591 (NR)	N=3322; of n=499 recruit-ed exc. n=192 inc. n=307 (NR)	n=88 (NR)	n=438 (87.6%)	n=795 (NR)	n=337 (36.6%) smokers (n=82; 8.9%)	n=1497 (99.8%)	n=697 (62.3%)	n=473 (NR)	n=82 in focus groups, n=32 inter-views	n=333 (NR)	n=2190 (73%)	n=1273 (NR)
8 Are the measurements (questionnaires) likely to be valid and reliable?	No	Unclear	Yes	Yes	No	No	Yes	Yes	Unclear	Yes	Unclear	Unclear	Unclear	Unclear	Unclear
9 Was the statistical significance assessed?	Yes	Yes	Yes	Yes	Yes	No	Yes	Yes	Yes	Yes	Yes	N/A	Yes	No	Yes
10 Are confidence intervals given for the main results?	Yes	Yes	Yes	Yes	Yes	No	Yes	Yes	Yes	No	Yes	N/A	Yes	Yes	No
11 Could there be confounding factors that haven't been accounted for?	No	No	No	No	No	Yes	No	Yes	No	No	No	Yes	No	No	No
12 Can results be applied to your organisation?	No	Yes	Yes	No	Yes	Unclear	Yes	Yes	Yes	Yes	Yes	Yes	Yes	Yes	Yes

Notes	Validated tool; Reasonable level of participation; Study is 24 years old but still holds some relevance	Not a validated tool; Recruitment methods questionable	Based on previous surveys but not clear if validated; Clear methods; High number of participants although response rate not reported; Well written	Validated tool; Large target population clearly described but low recruitment; ell written	Not a validated tool; Low participation level; N and n unexplained in tables	Not a validated tool; Limitations of the study not covered; Not clear who by and how potential participants recruited	Validated tool; High participant numbers; Limitations discussed	Validated tool; Clear methods; Very low number recruited	Not clear if validated tool; High participation; No missing data reported; Poorly written	Validated tool; Clear method of recruitment; Administrative staff data presented separately; Limitations discussed	Not a validated tool; Discrepancies in reported participation figures; Limitations discussed	High number of participants for focus group & interviews; Lack of current and former smokers amongst participants; Little data provided	Not clear if validated tool; Low participation; Considerable amount of missing data	Not a validated tool; High level of participation; Limitations discussed	Unclear basis of data collection tool; Large population clearly described; Unclear why random sample rather than whole population; No mention of missing data
GRADE defined quality of evidence (high, moderate, low, very low)	Low	Low	Low	Low	Very low	Very low	Moder-ate	Very low	Low	Moder-ate	Low	Very low	Very low	Low	Low

Supplementary Table S2. Data Extraction of included (n=10) and excluded (n=5) studies

Author et al (Publishing year)	Aim	Setting	Methodology & methods	Population	Sample size & response rate	Key findings	Notes
**Salih & Farghaly 1996**[9]	To throw more light on the psychosocial and behavioural aspects of smokers associated with participation, attrition and the outcome of smoking cessation programs	Anti-smoking center at a specialist hospital in Buraydah	Quantitative; Administered survey utilizing the United States Preventative Medicine Institute / Strang Clinic Health Action Plan during October 1994 to September 1995 with 6 month follow up;<br><br>No mention of ethical approval or consent for research	Smokers	N=326 selected by systemic random sampling, alternately on initial visit to enrol;<br><br>Response rate not provided	38.3% were described as quitters at time of follow up; chances of quitting significantly decreased with age; marital status, nationality, residence were not significant predictors; literacy was significantly indicated amongst quitters – the rate of success had been highest among the better educated; recommends smoking prevention education begins at an earlier age, particularly among persons of low socio-economic status; continuers had smoked more, for longer periods and found it more difficult to stop; 96% of quitters (against 47.8%) had a past history of attempts to stop also previous length of time stopped; assessment of patient's individual characteristics would allow physicians to target their efforts	Based on established tools modified and translated to Arabic although no mention of back-translation or piloting; although dated there is still commonality with content and outcomes of current studies
**Al-Mohrej et al 2014**[27]	To determine the prevalence of cigarette smoking and to predict the effect of price increase on cigarette consumption	Riyadh plus social media	Quantitative; Cross-sectional study April-May 2013 on paper distributed in malls and circulated on Twitter; not clear how tool developed; Sample size calculation n=2160 (not clear how the population estimated nor geographical control on social media);<br>No mention of ethical approval or consent	Saudi nationals	N=2160, including 103 inadmissible as non-Saudi	Authors reported: 'a striking' 39% of respondents were current smokers of whom 92% were male; 48.7% of non-smokers were male; smokers were aged 21-30 (55%), had a higher income and were more educated; smokers (56%) and non-smokers (58%) thought cigarettes were expensive; 61% of smokers thought an increase would not affect consumption while 74% of non-smokers thought it will decrease consumption; 55% of smokers and 83.5% of non-smokers thought a price of 8.27 US dollars would lead to smoking cessation; females, those who smoked most and those who thought the current price expensive were least in favour of the price increase; the price of a packet of cigarettes must triple to observe a significant reduction in consumption	Sample size calculation and response rate match exactly, taking account of inadmissible submissions (n=103) does not meet the required power; use of 'famous Saudi athletes Twitter account' to promote study

**Almutairi 2014a[15]**	To compare the prevalence of smoking among students, faculty and staff and examine their interest to quit To determine the difference on perceptions of smoking and non-smoking student, faculty and staff with regard implementation of a smoke-free policy	University in Riyadh	Quantitative; Cross-sectional self-administered survey in 2013 in part based on previous surveys; pilot tested and modified (no details); Ethical approval gained with verbal consent from participants	Adults over the age of 18 years students and staff randomly selected	n=4591 surveys returned with 11 excluded for missing data	Authors reported that: respondents were female (54.1%), aged 24 years of age or younger (74.6%), single (73.6%), smoking more prevalent amongst males (22.6%) than females (5.9%); staff had highest rate of smoking (36.7%); 67.4% of students who smoke (n=402) indicated that they try to quit smoking; quit attempts were not found to be significant; students and faculty were more likely to attempt to quit than staff; majority of respondents agreed that smoking cause harm due to second hand smoke; non-smokers were supportive of the smoking ban in public places as were most smokers; smokers were least likely to support fines and other disciplinary sanctions; smokers (81.5%) and non-smokers (94.2%) thought there should be more help or support from the clinics for people who want to quit smoking	Abstract has typo: 'is to compares' should be singular; The exclusion criteria are stated as the opposite of inclusion when exclusion should be subset of inclusions not eligible (for example, welfare or literacy reasons); The second aim is unnecessarily repeated in the Design and Setting section; inconsistent on whether the survey is (self-) administered; Main text typo 'amongstudents', 'there should fines', 'smoking were not bad', 'ismore', in the survey ''smoking should be ban'
**Al-Zalabani et al 2015[10]**	To determine the relevance and to identify factors affecting the 'intention to quit' among intermediate and secondary school current cigarette smoker students	Schools in Al Madinah city	Quantitative; Cross-sectional survey conducted in April-May 2013 based on GYTS (WHO 1998); Pre-tested, piloted; Ethical approval gained	Intermediat e and secondary school students who are current smokers	Three-stage cluster sampling based on schools (N=354), graders and classes to randomly select a representative sample; Students surveyed (n=3322) in schools (n=36); Response from current smokers (n=499) of which excluded for missing data/ inconsistent	Authors reported: respondents were male (n=239; 77.9%), age 17 and over (54.7%), living with parents (94.1%) and Saudi nationals (83.7%).Overall, 71.7% demonstrated intention to quit smoking within one year (males 75.7%; females 57.4%); 70.5% with an intention to quit made at least one attempt in the last year; reasons for quitting included preserving health and saving money; 87.9% believed that smoking is hazardous to health, 59.6% supported its ban in public areas; participants willing to quit were more likely to have non-smoking parents and friends, be exposed to second hand smoke less than 4 days per week, smoke fewer than 10 days per month, smoke fewer than 5 cigarettes per day, have received home and school messages regarding harmful effects of smoking;	Well written and clearly reported; addresses the research questions; Typos approximatly, Kingdome

					responses (n=192); Response rate not provided but n=301		
**Hajjar et al 2016[29]**	To determine the number of surgical patients who received education about the magnitude of smoking cessation prior to surgery by their treating surgeons, and to determine the number of surgeons who preoperatively inform their patients about the risk of smoking on surgical outcomes. IN addition, to assess the number of patients who agreed to quit smoking completely after surgery by evaluating patients' compliance with smoking cessation advise	University hospital, Riyadh	Quantitative; Descriptive, comparative cross sectional study using validated, pre-piloted self-administered questionnaires in January – March 2013; Ethics and consent not mentioned	Outpatients , inpatients, (and treating surgeons who were not the focus of this SR so are not reported here)	N=795 patients; Surgical patients (n=108) and non-surgical patients (n=687)	The majority of surgical (95.4%) and non-surgical (94%) patients agreed to quit smoking before surgery; 66.7% of surgical and 73.1% of non-surgical patients were unaware of the harmful effects of smoking; of the surgical patients, 58.8% declared they were advised to stop smoking before the surgery, 51.4% were advised to stop smoking after surgery; the advice on how long before surgery to stop varied from 1 week (41.2%) to more than 4 weeks (17.6%); and post-surgery, for 4 weeks for better health (50%); 70.6% of patients were willing to quit smoking after their surgery	Generally well reported with survey tools provided; discussion on limitations included; recommendations and conclusion based on findings
**Abdelwahab et al 2016[21]**	To study cigarette smoking patterns and to evaluate sociodemographic associates of cigarette smoking and cessation behaviors of smokers	Public Healthcare Centres (PHC) in Jazan region	Quantitative; Cross-sectional study including interviews and questionnaire; not clear if validated tool; pilot data not included in analysis Ethical approval gained plus written consent	Randomly selected Saudi citizens, both smoking and non-smoking, aged 15 years or older attending PHC	N=1497; Response rate 99.8%	Author reported: respondents mainly male (female 1.5%), married, university educated, employed and younger than 34 years old; 49.2% were smokers; life tensions and peers influenced smoking; 88.6% felt that school awareness programs are potentially successful; independent predictors of smoking were geographic area, gender, marital status, education, job and age; the majority planned to quit or had previously tried to quit	Exceptionally high response rate particularly for a sensitive invasive study; describes sociodemographic uniqueness which suggests not generalisable; spelling (researches, comminutes, variable); in the abstract, 'more than 50% of the study sampled had tried at least once to quit

							smoking' – suggest this is 50% of the smokers
**Mahdi et al 2018**[31]	To estimate the magnitude of tobacco smoking among healthcare workers and to assess the need to establish a smoking cessation program	Public, tertiary healthcare organization in Makkah	Quantitative; Cross-sectional, self-administered, online based on WHO/CDC Global Health Professional Survey; June – September 2015; IRB ethical approval	Day shift	n=697 (62.3%); stratified random sample based on physicians and allied healthcare workers (neither reported as match exclusion criteria) and administrative staff	The authors report that: most participants were aged 19-30 years (44.5%); male (62.3%; Saudi (45.6%), current smokers (18.4%) of whom low nicotine dependence (46.9%), former smokers (9.8%); administrative staff (n=201) current smokers (n=61; 30.3%) are significantly more likely to smoke, should be the focus of smoking cessation programs; no significance of educational level, shift working or age; smoking HCW (80.3%) positive attitude towards smoking cessation; smokers agreed (71%) that healthcare professionals serve as role models, smokers willing to quit (65%)	Includes administrative workers (reported) as well as healthcare professionals (match exclusion criteria); validated data collection tool; large, stratified sample including representative proportion of smokers and former smokers
**Onezi et al 2018**[32]	To investigate the use of extra-treatment, in the form of social media support groups, for preventing smoking relapse	Hospital clinic and PURITY (Saudi anti-smoking association) both in Riyadh	Quantitative; Cross-sectional survey administered face-to-face; Ethical approval gained plus written informed consent	Smokers who expressed interest in quitting and attempting to quit	n=473 convenience sample; 3 groups: Twitter based support (n=150); WhatsApp based support (n=150); control group offered telephone support (n=173) Response rate not provided but group figures given as Twitter 24%, WhatsApp 35%, control 41%	The authors report that: those using the social media support had reduced smoking frequency (42%), had not reduced (6%), believed likely to reduce in future (52%); 75% satisfied with social media support; comparison of the 3 groups smoking reduction reports outcomes are unclear. Use of each social media was favoured over not using social media, at one point favouring Twitter over WhatsApp, then comparing all 3 groups 'less difference was found between those subscribed to social media than between those not subscribed'; Recommendations are: 'to train the people responsible for developing and administrating such support groups' also 'those struggling to quit smoking, should be encouraged to join support groups on the social media platform of their choice'	Discrepancy between figures for recruitment and participation (n=150/150/173; 24/35/41%) unexplained; some possible mis-translation of language 'alleged', 'assumed'; there is a typo in Figure 1 spelling of WhatsApp; the % in Figure 2 do not match the textual description; over 50% missing data for several questions
**Al-Nimr et al 2020**[35]	To describe the characteristics of female Saudi smokers and their self-reported reasons for starting to smoke	Clinics offering smoking cessation programmes across	Quantitative; Cross-sectional self-administered survey conducted from January	Female smokers	N=3000 first time attendees at 18 clinics; n=2190 (73%)	The authors reported that: 67.4% of women reported the presence of another smoker at home; the most common reason for starting to smoke was friends who smoke (31.1%); the most common reason cited in terms of willingness to	Not based on validated tools nor piloted. However, fully described and detailed on both data collection, survey tool content and

	and willingness/ unwillingness to quit smoking	Saudi Arabia	2014-January 2017; Written informed consent and ethical approval gained			quit smoking was health concerns (45.5%); the most cited reason for being unwilling to quit smoking was fear of mood changes (28%); the authors note it would have been interesting to ask specifically about whether pregnancy was a reason considered to stop smoking. Key recommendation is introduction of on-campus smoking cessation clinics at colleges and integration of referral as part of routine clinical care	data analysis; missing data accounted for and CI provided
**Amin et al 2020[36]**	To determine the prevalence of tobacco smoking (main text also water pipe) in healthcare students along with environmental exposure and potential influential factors	University of Riyadh	Quantitative; Cross-sectional self-administered survey in January-April 2019; validated by two staff; pilot tested 20 healthcare students, no mention of outcome of pilot; Ethical approval from University IRB; consent 'process was clear' but not expressly captured	Randomly selected first to fifth year healthcare students based on year, gender from university provided list	n=1273	Authors reported that: prevalence of tobacco smoking was 13.7%; males (20.6%), females (2.5%); highest on College of Applied Sciences (34.5%) lowest in Pharmacy (10.9%); 18-21 year olds (43.1%), 22-25 years (51.1%), 26 and older (5.8%); reasons for smoking cigarettes were fun or passing time (45.2%), relieving stress (33.3%); water pipe smokers (12.5%) to enjoy its flavours (42.1%); second hand smoke (31.7%), environmental smoke (42.5%); tobacco users at home (female 51.2%; males 48.8%); primary care clinicians are first advisors on harms of smoking, reasons not to start smoking, importance of abstinence from tobacco use, counselling interventions to aid cessation in all age groups including parents; question environmental factors and cultural norms which promote smoking; train students in stress relief, add smoking cessation to curricula, improve access to effective quitting treatments, cigarette price increases and media campaigns	Inconsistent aim between Abstract and main text; some of the recommendations are not clearly linked to the data collected
**Excluded studies (n=5)**							
**Alyamani et al 2016[28]**	Abstract: To assess the prevalence and predictors for smoking cessation among undergraduate and	Private medical college, Riyadh	Quantitative; Cross-sectional survey in February – April 2016; Ethical approval gained	2nd to 6th year medical students currently smokers or quit during	n=88; no response rate provided	Authors reported: participants were mostly male (72.7%), aged below 25 (83.1%), in 5th year (29.5%); 21.6% have quit smoking during medical school; 65.9% were daily smokers; age of onset was 15-21 years (68.2%); smoking cessation was associated with increased	Neither N nor n explained in Tables; very small numbers on which to base findings; a lot of typos; discussion includes 'resluts not presented in

	graduate students of medical college; Introduction: Estimated the prevalence of smoking quitters among undergraduate and graduate medical students and analysed correlation between acquiring medical knowledge of the harmful effects of smoking and the smoking cessation decision. In addition, further predictors of smoking cessation during medical school were analysed.			medical college; recruited through an announcement and flyers to be completed during working hours or by telephonic interview		age and increased academic level; ex-smokers were more likely to have made several attempts to quit;	Tables'; non-academic language and style, for example, 'actions warranted to fight against medical students smoking'; 'etc…'
**Baig et al 2016**[13]	To evaluate the reasons and motivations towards cigarette smoking and barriers against quitting smoking and explored their knowledge and attitudes towards smoking	University medical faculty and general area in Jeddah	Quantitative; Cross sectional self-administered survey based on previously published related studies; Informed consent taken; Ethical approval gained	Young smokers amongst medical faculty students and general population	N=500 randomly selected; 87.6% (n=438)	Authors reported in frequency tables that: 61% of respondents started smoking aged 15-19; 60.7% smoked 11-30 cigarettes per day; 42.9% were motivated to smoke by their friends while 33.8% could not identify a motivational factor; smoking relieved tension and anxiety (37.2%), relieved boredom (28.8%); 73.3% wanted to quit smoking; 94.3% acknowledged smoking is harmful to health. Concluded: knowledge and attitude about smoking were good, and the majority of smokers were well aware of the associated hazards; there is a need to search out ways and means to help them to quit this addiction.	Gender is not reported but several mentions of 'boys'; only male relatives listed in questions; there is inconsistency in describing the participants as 'being university students' not general population; incorrectly presents combined figures from two questions ('smoking is a sign of maturation', 'smoking helps to mix in social gatherings') as totals; refers to respondents as 'youngsters' even though mean age is 22.89; question format restricted to single response when multiple more appropriate e.g. reasons for not quitting

							smoking, motivation for smoking; speculation not based on findings in discussion on pricing of cigarettes; no study limitation provided
**Almogbel et al 2016[30]**	Abstract: To determine the predictors of willingness to quit smoking among a cohort of male Saudi students<br>Main text differs: sample of male college students	3 Higher education institutes	Quantitative; Cross-sectional self-administered pre-tested (test-retest), validated (face, content) questionnaire in December 2011-January 2012; Ethical approvals gained; written informed consent from participants	Medical and non-medical student smokers	Convenience sampling of (N=70 000) students; questionnaire distributed in lecture for 20 minutes allowed and drop box;<br>920 surveys distributed, 467 returned, 130 excluded missing data; Response rate reported as 36.6% (smokers only response rate 8.9%)	Authors reported: 65% (n=53) of smokers thinking about quitting; average age of smokers was 22.1 +- 2.2 years; age started smoking 15 +- 4.7; significant impact on willingness to quit smoking were one or more previous attempts, seen anti-smoking messages on TV, or in the newspaper; higher addiction level associated with lower willingness to quit smoking	Abstract states administered survey while main text is self-administered;<br>Data analysis tool SAS 9.3 lacks full reference;<br>The response rate should be based on smokers only (n=82/920; 8.9%) and only smokers reported as no attempt to establish whether non-smokers had formerly smoked; addiction level reported in Discussion but not in Results yet was a large part of the description in Methods section; missing data not accounted for in Table of characteristics; no breakdown in Results of (non-)medical student smokers; only previous quit attempts reported as significant in Conclusion (Results noted anti-smoking as significant)
**Jradi & Saddik 2018[19]**	This study aims at exploring people's awareness and perceptions on cigarette health warning labels and assessing the impact of those labels on adult smoking behaviour	University hospital campus, Riyadh	Qualitative; Cross sectional focus groups plus face-to-face semi-structured interviews; January-August 2015;	Health-care providers, adult men and women and community leaders (Imams)	N=82 convenience sample for focus groups plus N=32 for interviews; Focus groups (n=9): health-care providers	Focus group participants were: current smokers (10%), former smokers (8.8%); mostly aware of the presence of health warnings/graphics on cigarette packages; most did not recall reading the warning messages; awareness levels of the specific details were low; consensus that current labels do not seem to be effective; full support for placing health warning labels on tobacco products;	The study is about the impact of labels on smoking behaviour but very few current or former smokers took part. Why was it important to gather the views of non-smokers? 10 of the 21 current or former smokers were

			Ethical approval gained plus written consent		(n=28), women (n=30), men (n=24); Imam interviews (n=32)	Community leaders expressed similar views and added their disapproval of the revealing body imagery on labels from other countries; 'bad company' was given as the main reason people smoke	healthcare providers with only 1 former and no current smokers in the women only focus groups; very little of the transcribed focus group extracts is provided
**Alqurashi et al 2019**[34]	To identify the prevalence of quitting rate within 4 months, the predictors of quitting status, and the prevalence of quitting attempts	Anti-smoking clinics at the primary health care centres in Jeddah	Quantitative; Retrospective review of records followed by survey administered by telephone at one year follow up in 2018; Verbal consent for interview with immediate participation	Smokers enrolled in the anti-tobacco program in 2017	N=333 at 3 out of 6 main smoking cessation clinics; 100% response rate inferred but considerable amount of missing data	Authors reported that: 86.5% were non-quitters; those who tried to quit tend not to last longer than 4 months; patients who started smoking at an earlier age than 16 years old were most likely to negatively influence smoking cessation; no other significant association of characteristics was found of predictors of smoking cessation; recruitment among female smokers must be promoted	Not clear: what constitutes a main clinic; how the random selection of 3/6 clinics was done; piloted with 10% at named 1 of the 3 clinics with pilot data not included in full study yet N not reduced; considerable amount of missing data (1 question 51% not filled) with only gender and smoking status completed by all
